# Xenotransplantation of Mitochondrial Electron Transfer Enzyme, Ndi1, in Myocardial Reperfusion Injury

**DOI:** 10.1371/journal.pone.0016288

**Published:** 2011-02-14

**Authors:** Cynthia N. Perry, Chengqun Huang, Wayne Liu, Najib Magee, Raquel Sousa Carreira, Roberta A. Gottlieb

**Affiliations:** 1 Department of Pathology, University of California San Diego School of Medicine, La Jolla, California, United States of America; 2 SDSU Bioscience Center, San Diego, California, United States of America; Instituto de Química - Universidade de São Paulo, Brazil

## Abstract

A significant consequence of ischemia/reperfusion (I/R) is mitochondrial respiratory dysfunction, leading to energetic deficits and cellular toxicity from reactive oxygen species (ROS). Mammalian complex I, a NADH-quinone oxidoreductase enzyme, is a multiple subunit enzyme that oxidizes NADH and pumps protons across the inner membrane. Damage to complex I leads to superoxide production which further damages complex I as well as other proteins, lipids and mtDNA. The yeast, *S. cerevisiae*, expresses internal rotenone insensitive NADH-quinone oxidoreductase (Ndi1); a single 56kDa polypeptide which, like the multi-subunit mammalian complex I, serves as the entry site of electrons to the respiratory chain, but without proton pumping. Heterologous expression of Ndi1 in mammalian cells results in protein localization to the inner mitochondrial membrane which can function in parallel with endogenous complex I to oxidize NADH and pass electrons to ubiquinone. Expression of Ndi1 in HL-1 cardiomyocytes and in neonatal rat ventricular myocytes protected the cells from simulated ischemia/reperfusion (sI/R), accompanied by lower ROS production, and preservation of ATP levels and NAD+/NADH ratios. We next generated a fusion protein of Ndi1 and the 11aa protein transduction domain from HIV TAT. TAT-Ndi1 entered cardiomyocytes and localized to mitochondrial membranes. Furthermore, TAT-Ndi1 introduced into Langendorff-perfused rat hearts also localized to mitochondria. Perfusion of TAT-Ndi1 before 30 min no-flow ischemia and up to 2 hr reperfusion suppressed ROS production and preserved ATP stores. Importantly, TAT-Ndi1 infused before ischemia reduced infarct size by 62%; TAT-Ndi1 infused at the onset of reperfusion was equally cardioprotective. These results indicate that restoring NADH oxidation and electron flow at reperfusion can profoundly ameliorate reperfusion injury.

## Introduction

Mammalian NADH-quinone oxidoreductase (complex I) is a 900kDa mitochondrial enzyme made up of at least 45 individual subunits [1]. It is responsible for the oxidation of NADH, contributes to the formation of the proton gradient which drives ATP synthesis, and passes electrons to ubiquinone in the respiratory chain [2]. Heritable disorders involving complex I include myopathies, Parkinson's Disease, Mitochondrial encephalopathy with lactic acidosis and stroke-like syndrome (MELAS), and Leber's hereditary optic neuropathy (LHON) [3]. Ischemia/reperfusion (I/R) injury is characterized by decreased complex I respiration [4] and increased formation of reactive oxygen species (ROS) [5], [6], which in turn induce further damage to complex I, lipids, mtDNA, mitochondrial proteins and other cellular targets. ROS-induced ROS release can trigger catastrophic opening of the mitochondrial permeability transition pore (mPTP) [7].

In bacteria and fungi, the enzymatic function of mammalian complex I is carried out by structurally simpler enzymes, collectively named NDH-2, which oxidize NADH and act as the entry site of electrons to the respiratory chain [8], [9]. The *S. cerevisiae* mitochondrial NADH-quinone internal oxidoreductase (Ndi1), a single polypeptide enzyme, oxidizes NADH and transfers electrons to ubiquinone, but unlike complex I, it does not pump protons across the inner membrane. Located on the matrix side of the inner mitochondrial membrane, Ndi1 is a 513aa, 56kDa protein containing a noncovalently bound FAD. Ndi1 is insensitive to complex I inhibitors rotenone and 1-methyl-4-phenylpyridium ion (MPP^+^) but is sensitive to inhibition by flavone [9]–[11]. Much interest has arisen in the ability to complement dysfunctional mammalian complex I with Ndi1 or related enzymes from other simple organisms.

Previous work by Yagi et al. showed that Ndi1 could be expressed in human cells and could function in parallel with complex I [12], [13]. Ndi1 expression was sufficient to restore respiratory activity in complex I deficient Chinese hamster CCL16-B2 cells [14] and to protect against neurodegeneration in an MPTP-induced mouse model of Parkinson disease [15]. In the present study, we sought to determine whether Ndi1 delivered by protein transduction could provide cardioprotection in *in vitro* and *ex vivo* models of ischemia/reperfusion.

## Materials and Methods

### Cloning

Construction of the mammalian expression vector pHook(Ndi1) has been previously described [14] and was a generous gift from Akemi and Takao Yagi (The Scripps Research Institute, La Jolla, CA). To create TAT-Ndi1, the full length NDI1 insert was amplified using 5-GCTT*GGTACC*AGTTTCATCAC-3 (KpnI site italicized) and 5-GGC*GAATTC*TCAAGCATCATTTCG-3 (to generate a new EcoR1 site, italicized). This product was inserted into the 6xHis-TAT-HA cloning vector (pTAT-HA, where HA is hemagglutinin) kindly provided by Dr. Steven Dowdy (UCSD, La Jolla, CA). Both the TAT and HA epitope are located N-terminal to the insert which contains a 26aa N-terminal mitochondrial targeting sequence original to yeast *S. cerevisiae*.

### Cell culture and sI/R

The HL-1 cardiomyocyte line (generous gift of W. Claycomb) was maintained in Claycomb media as previously described [16]. Cells were co-transfected with pHook-Ndi1 and mitochondrially targeted DsRed (pDsRed2-mito, Clontech, Mountain View, CA) and then subjected to simulated ischemia and reperfusion (sI/R). Neonatal rat ventricular cardiomyocytes (NRVM) were prepared as previously described [17] and plated on gelatin-coated dishes. Transient lipid-based transfection of plasmid DNA was performed using Effectene transfection reagent (Qiagen) per manufacturer's recommendation. NRVM and HL-1 cells were transfected at 60% confluency and used 48 hr later. sI/R was performed by buffer exchange from Krebs-Henseleit (KH, in mM: 110 NaCl, 4.7 KCl, 1.2 KH_2_PO_4_, 1.25 MgSO_4_, 1.2 CaCl_2_, 25 NaHCO_3_, 15 glucose, 20 HEPES, pH 7.4) to ischemia-mimetic solution (in mM: 125 NaCl, 8 KCl, 1.2 KH_2_PO_4_, 1.25 MgSO_4_, 1.2 CaCl_2_, 6.25 NaHCO_3_, 5 Na-lactate, 20 HEPES, pH 6.6) and placing the dishes in hypoxic pouches (GasPak™ EZ, BD Biosciences). After 2 hr, reperfusion was initiated by return to room air and buffer exchange to normoxic Krebs–Henseleit solution. Controls incubated in normoxic KH solution were run in parallel for each condition and showed no loss of cell viability. Cell death was scored by Yo-Pro-1 (Molecular Probes) staining (a marker for loss of membrane integrity) and imaged by fluorescence microscopy. Greater than 200 cells were scored per condition for cell death assay and each experiment was performed at least three times. To detect ROS production, CM-H_2_DCFDA (10µM, Invitrogen) was added to cells grown in 96-well plates and incubated for 30 min. Quantification was done by fluorescence plate reader (excitation/emission 490/520nm). Isolation of adult rat cardiomyocytes was performed as previously described [18]. Briefly, rat hearts were perfused with heart media (10mM HEPES, 30mM taurine, 2mM carnitine, 2mM creatine in JMEM) for 4 min at 3 ml/min and then digested with digestion buffer (1 mg/ml of collagenase II, 6.25 µM CaCl_2_ in 50 ml perfusion buffer) for 18 min at 3 ml/min. The heart was then removed and minced in digestion buffer, to which stop buffer (perfusion buffer containing 12.5 µM CaCl_2_ and 5% newborn calf serum) was added. Cells were allowed to sediment by gravity for 8–10 min in a 50-ml Falcon tube. The supernatant was removed, and the pellet was resuspended in 30 ml of room temperature stop buffer. Calcium was then reintroduced to myocytes gradually to achieve a concentration of 1 mM, while being monitored by microscopy. Rod-shaped myocytes (100,000 per 2 ml) were plated in laminin-coated 35-mm dishes and allowed to recover overnight. Where indicated, TAT-Ndi1 was transduced into adult myocytes or NRVMs by addition to media at 500nM and incubated 20 min prior to sI/R treatment. TAT protein was removed with buffer exchange. Adult cardiomyocytes were subjected to 2 hours simulated ischemia and 2 hours reperfusion as described above.

### Preparation of heart lysates

Frozen heart samples were thawed on ice in homogenization buffer (In mM: 50 Tris-HCl pH 7.4, 1 EDTA, 1 EGTA, 150 NaCl, 1 PMSF, 0.01 leupeptin, 0.01 E-64, and 1% Triton-X 100) [40]. The tissue was minced and Polytron homogenized (Kinematica, Basel, Switzerland) at 0°C with 3 pulses of 5 sec each. The homogenates were centrifuged at 3,000×g for 10 min at 4°C to remove nuclei and cell debris. The supernatant, designated as heart homogenate, was aliquotted and stored at −80°C until use.

### Preparation of isolated mitochondria

Hearts were rapidly excised and ventricles were minced and homogenized twice by polytron for 2.5 sec in ice-cold mitochondrial isolation buffer (MIB, in mM: 10 MOPS pH 7.4, 250 sucrose, 5 KH_2_PO_4_, 2 MgCl_2_, 1 EGTA, 0.1% essentially fatty acid-free BSA). Lysates were centrifuged twice for 5 min at 600×g to remove unbroken tissue and nuclei and the supernatants were centrifuged for 10 min at 3,000×g to pellet mitochondria. The crude mitochondrial pellet was resuspended in swelling buffer (MIB without BSA supplemented with 5 mM EGTA, 5 mM pyruvate, and 5 mM malate).

### Preparation of submitochondrial fractions

Crude mitochondria (prepared as above) were resuspended in 20 mM Hepes/PBS with fresh protease inhibitors and sonicated for three cycles of 10 seconds each, then centrifuged at 120,000×g for 3 hours at 4°C. The supernatant, containing the soluble mitochondrial proteins, was concentrated using a Microcon IM-10 spin column (Millipore, Billirica, MA). The membrane pellet was washed in 20 mM Hepes/PBS and centrifugation repeated. The final membrane pellet was resuspended in homogenization buffer containing 1% Triton-X 100 (as above).

### Mitochondrial swelling assay

60 µg of isolated mitochondria were suspended in 200µL swelling buffer in a 96-well plate. Where indicated, TAT-Ndi1 (500nM) or flavone (0.5mM) were added and Ca^2+^ (250 µM) was used to induce swelling. Absorbance was monitored in a plate reader at 520 nm for 45 min at room temp.

### Mitochondrial Respiration

200µg mitochondria were added to KCl respiration buffer (In mM: 140 KCl, 10 MgCl_2_, 10 MOPS pH 7.4, 5 KH_2_PO_4_, 1 EGTA, 0.2% essentially fatty acid-free BSA). Oxygen consumption was recorded polarographically at 25°C using a Clark-type oxygen mini-electrode (Hansateck, UK) in a water-jacketed reaction chamber. Palmitoyl-L-carnitine (40µM), malate (2.5mM), ADP (1mM), rotenone (0.5µM), and flavone (50µM) were added to respiration chamber sequentially at indicated time points.

### Western blot analysis

Proteins prepared from rat hearts and cultured cells were quantified by Bio-Rad protein assay. For immunodetection, 50 µg of heart lysate prepared as above was resolved on SDS-PAGE 10–20% denaturing gels and transferred to PVDF nylon membranes. The membranes were blocked with 5% nonfat dry milk in 1× TBST buffer (100mM NaCl, 10mM Tris-HCl (pH 7.4), and 0.1% Tween-20) for 1 hr, then incubated with 500-fold diluted monoclonal primary antibody against HA (Santa Cruz) at 4°C overnight, washed with TBST buffer at room temp, and incubated with goat anti-mouse horseradish peroxidase-conjugated secondary antibody (1∶2000 dilution). Immunoreactive bands were visualized by chemiluminescence (Super Signal West Dura Substrate, Pierce). Each immunoblotting experiment was repeated three times unless otherwise indicated and the results were averaged.

### Langendorff perfusion

Isolated rat hearts were perfused in Langendorff mode as previously described [19], [20]. In brief, after anesthesia and heparinization (pentobarbital sodium 60 mg/kg i.p. and heparin 500 U i.p.), rat hearts were excised into ice-cold Krebs-Ringer buffer (KRB) and within 30s were retrograde perfused via the aorta with oxygenated buffer. Hearts were allowed to stabilize at constant pressure (60 mm Hg) 20 min prior to I/R and where indicated, TAT-Ndi1 (500 nM) was added to the perfusion buffer for 15 min before ischemia or at the onset of reperfusion. Global no-flow ischemia was maintained for 30 min and reperfusion was accomplished by restoring flow with oxygenated buffer for 15 min (for all measurements except infarct size determination). CK release was quantified with the CK EC 2.7.3.2 UV test kit (Stanbio Lab, Boerne, TX). In brief, the coronary elute was collected from the first 15 min of reperfusion, pooled and 1mL aliquots were used for analysis according to manufacturer's recommendations. Ongoing production of superoxide in heart slices after the perfusion protocol was quantified by measuring ethidium fluorescence derived from oxidation of dihydroethidium as described [21], [22]. Relative ATP levels were quantified by luminescent plate reader using Cell Titer Glo kit as recommended (Promega, Madison, WI). Lipid peroxidation was determined by measuring malondialdehyde (MDA) and 4-hydroxyalkenals (HAE) levels according to manufacturer's instructions (Oxford Biomed, Oxford, MI). NAD+/NADH ratios were determined by colorimetric plate reader assay per manufacturer's specifications (Biovision Inc., San Francisco, CA). Biochemical analyses of ischemic and reperfused heart tissue were performed on hearts quick-frozen in liquid nitrogen. Infarct size determination by triphenyl tetrazolium chloride (TTC) staining was performed on hearts reperfused for 120 min [23]. 2mm tissue sections were stained and a minimum of 5 sections per heart were analyzed. Volume analysis of infarct size was performed using Adobe Photoshop (Adobe Systems, San Jose, CA). All procedures were approved by the Animal Care and Use Committee at San Diego State University and conform to the *Guide for the Care and Use of Laboratory Animals* (National Institutes of Health publication no. 85-23, revised 1996).

### TAT-mediated protein transduction

Recombinant protein expression and purification was performed as described [24]. Briefly, a 2mL LB-ampicillin (100 µg/ml) culture of bacteria transformed with pTAT-Ndi1 was grown at 37°C and 225 rpm for 5 hours and transferred to a 100mL overnight culture until it reached an OD_600_ of 0.9–1.2. The overnight culture was diluted into a final volume of 1 L of fresh LB-ampicillin and incubated to an OD_600_ of 0.6–0.9. Isopropylthiogalactoside (IPTG, Roche) was added to the culture and incubated for an additional 10 hours at 130 rpm, 35°C. The bacterial pellet was harvested by centrifugation at 6000 rpm for 10 min at 4°C and resuspended in 15 mL of cold PBS. This was repeated and the final pellet dissolved in 15 mL buffer Z (8 M urea, 100 mM NaCl, and 20 mM Hepes, pH 8.0) plus 10mM imidazole. The lysate was sonicated on ice (3 pulses of 40 s) followed by centrifugation at 16,000 rpm for 30 min at 4°C. 7 mL of supernatant was applied to a 25 mL column packed with 6 mL of Ni-NTA resin (Qiagen) equilibrated in buffer Z plus 10 mM imidazole, rocked at 4°C overnight. The flow-through was collected by gravity flow and re-applied to the column twice. The column was washed with 50 mL of buffer Z and proteins were eluted in 17 mL buffer Z containing 250 mM imidazole followed by another elution with 17 mL buffer Z containing 1 M imidazole. Both elution fractions were pooled and concentrated to half the volume using an Amicon Ultra centrifugation device (Millipore). Immediately prior to use, 2.5 mL of eluate was desalted on a PD-10 column (GE Healthcare), equilibrated in PBS +10% glycerol and eluted with 3.5mL. TAT-Ndi1 was diluted into Krebs-Ringer Buffer (500nM final concentration). TAT-mediated protein transduction provides significantly higher efficiency, nearly 100% in cell culture, as compared to lipid-based transient transfection which has ∼40% efficiency; TAT-proteins allow for tissue-transduction as well. We previously showed that TAT-β-galactosidase control protein had no effect on heart function or infarct size [25]–[27], therefore, we did not include a TAT-β-gal control protein in the present studies.

### Widefield fluorescence microscopy

Cells and tissues were observed through a Nikon TE300 fluorescence microscope (Nikon) equipped with a 4× lens and a 20× lens (0.3NA, Nikon), a 60× Plan Apo objective (1.3 NA oil immersion lens; Nikon), a Z-motor (ProScanII, Prior Scientific), a cooled CCD camera (Orca-ER, Hamamatsu) and automated excitation and emission filter wheels controlled by a LAMBDA 10-2 (Sutter Instrument) operated by MetaMorph 6.2r4 (Molecular Devices Co.). Fluorescence was excited through an excitation filter for FITC (HQ480/x40), Texas Red (D560/x40). Fluorescent light was collected via a polychroic beamsplitter 8(61002 bs) and an emission filter for fluorescein isothiocyanate (HQ535/50m), and Texas Red (D630/60m). All filters were from Chroma. Acquired wide field Z-stacks were routinely deconvolved using 10 iterations of a 3D blind deconvolution by MetaMorph 6.2r4. Unless stated otherwise, representative images shown are maximum projections of Z-stacks taken with 0.20µm increments capturing total cellular volume.

### Statistical analysis

The probability of statistically significant differences between two experimental groups was determined by both paired Student's *t*-test and ANOVA. Values are expressed as means ± SD of at least three independent experiments unless stated otherwise, and *P* values are reported for ANOVA. A value of *P*<0.05 was considered significant.

## Results

### Ndi1 localizes to mitochondria and prevents cell death following simulated ischemia/reperfusion

**Figure 1:**
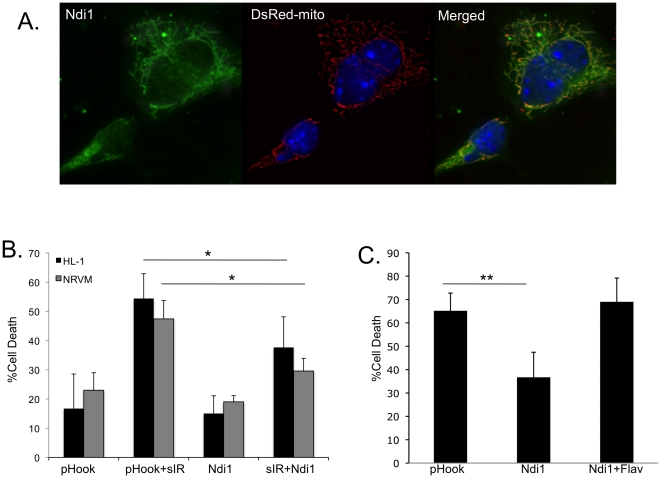
Ndi1-mediated cytoprotection following simulated ischemia reperfusion is specific. **A**. pHook(Ndi1) and pDsRed2-mito were transiently transfected into HL-1 cells. Ndi1 (stained with anti-HA antibody) co-localized with mitochondria (DsRed-mito) (merged). **B**. Ndi1-transfected and empty pHook-transfected HL-1 cells and neonatal rat cardiomyocytes were subjected to 2 hours simulated ischemia and 24 hours reperfusion. Cell death was scored by permeability to Yo-Pro-1 stain and only transfected cells were scored. (≥250 cells scored per experiment, n = 3, *p<0.05). **C**. Neonatal rat cardiomyocytes transfected with empty pHook or pHook(Ndi1) were subjected to 2 hours simulated ischemia and 24 hours reperfusion. Pretreatment with Ndi1-inhibitor flavone abolished the cytoprotective effect of Ndi1 expression. Cell death was scored by permeability to Yo-Pro-1 stain and only transfected cells were scored. (≥250 cells scored per experiment, n = 3, **p<0.005).

To confirm Ndi1 localization, HL-1 cells were transiently co-transfected with pHook(Ndi1) and pDsRed2-mito and Ndi1 was detected by immunostaining for the HA tag. Ndi1 co-localized with mito-DsRed with distinctive mitochondrial morphology (Figure 1A). To determine the ability of Ndi1 to protect against sI/R-induced cell death, transfected HL-1 cells and NRVMs were subjected to 2 hour simulated ischemia and 24 hours reperfusion. Expression of Ndi1 decreased cell death in HL-1 cells and NRVMs (Figure 1B). Pretreatment with the Ndi1-inhibitor, flavone, abolished cytoprotection in HL-1 cells subjected to sI/R (Figure 1C), indicating protection against cell death was due to Ndi1 activity.

### TAT-Ndi1 enters cardiomyoctes and localizes to mitochondria

**Figure 2:**
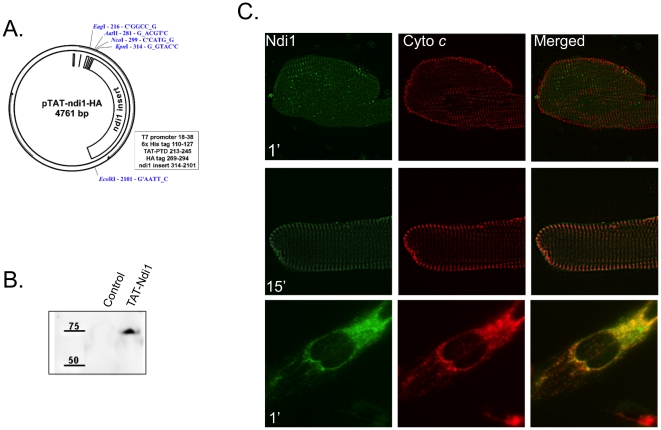
Generation of TAT-Ndi1 and expression *in vitro*. **A**. Map of TAT-Ndi1 construct generated from inserting full length NDI1 gene (1,539bp) from pHook(NDI1) into the 6xHis-TAT-HA cloning vector. **B**. Lysates of adult rat ventricular myocytes were transduced with TAT-Ndi1 at 500nM in complete maintenance media for 20 min. Cell lysates were probed with anti-HA antibody to detect TAT-Ndi1. **C**. Adult cardiac myocytes (first and second rows) and HL-1 cells (third row) were transduced with TAT-Ndi1 at 500nM for 1 or 15 min as indicated, fixed and double-labeled with affinity-purified rabbit antibody to *S. cerevisiae* Ndi1 and mouse monoclonal cytochrome *c* antibody.

To explore the possibility that Ndi1 might be useful in the context of I/R, we generated a cell-permeable recombinant protein consisting of full length Ndi1 fused to a hemagglutinin (HA) epitope and the undecapeptide protein transduction domain (PTD) of HIV TAT (TAT-Ndi1, Figure 2A). Adult rat cardiomyocytes were transduced with TAT-Ndi1 (Figure 2B&C) and detected by immunostaining for Ndi1. TAT-Ndi1 was detected in 100% of cells and displayed a mitochondrial morphology in >75% of myocytes, co-localizing with mitochondrial protein cytochrome *c* within 1 min in HL-1 cells and adult cardiomyocytes (Figure 2C).

### TAT-Ndi1 is protective against sI/R and remedies energetic deficits and oxidative stress in cardiac cells

**Figure 3:**
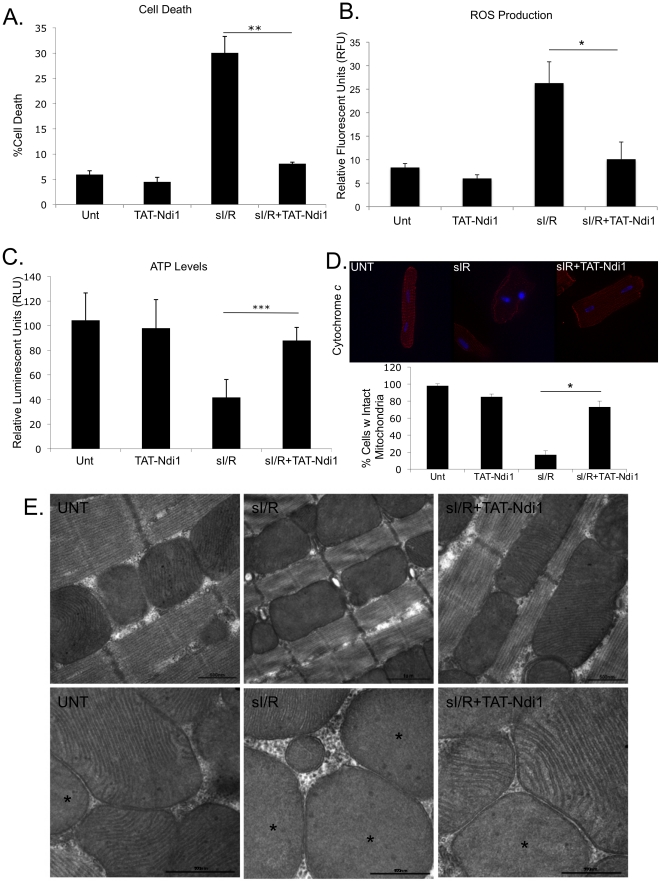
TAT-Ndi1 protects against sI/R and remedies energetic deficits and oxidative stress in cardiac cells. **A**. Neonatal rat cardiomyocytes transduced with 500nM TAT-Ndi1 for 20min were subjected to 2 hours simulated ischemia and 24 hours reperfusion. Cell death was scored by permeability to Yo-Pro-1 stain relative to total cell number determined by Hoechst 33342 staining. (≥250 cells scored per experiment, n = 3, *p<0.05). **B**. NRVMs were incubated 20 min with TAT-Ndi1 and subjected to 2 hours simulated ischemia and 24 hours reperfusion. Dichlorodihydrofluorescein diacetate was used to measure ROS levels in cells following 2 hours simulated ischemia and 24 hours reperfusion. (n = 3, *p<0.05). **C**. ATP production in control and TAT-Ndi1 transduced NRVMs following 2 hours simulated ischemia and 24 hours reperfusion. (n = 3, ***p<0.0005). **D**. Cultured adult rat cardiac myocytes were incubated with TAT-Ndi1 and then subjected to 2 hours simulated ischemia and 2 hours reperfusion. Cytochrome *c* release was detected by mouse anti-cytochrome *c* antibody and cells with intact mitochondria quantified by fluorescence. (≥100 cells scored per experiment, n = 3, *p<0.05). **E**. Adult rat cardiomyocytes incubated 20 min +/− TAT-Ndi1 were subjected to 2 hours simulated ischemia and 2 hours reperfusion. EM images are representative of two separate experiments. Asterisks denote mitochondria which have lost cristae architecture.

To confirm the cytoprotection results observed with transient transfection, NRVMs exposed to TAT-Ndi1 were subjected to sI/R. Cell death indicated by permeability to Yo-Pro-1 decreased from 30.0% to 8.1% in the presence of TAT-Ndi1 (Figure 3A). Oxidative stress, reduced ATP synthesis, and failure to oxidize NADH are results of complex I damage after ischemic injury. DCFDA was used to measure H_2_O_2_ production in NRVMs subjected to sI/R. Ndi1 reduced ROS production by 51% (Figure 3B), and preserved ATP levels after sI/R (Figure 3C). To detect mitochondrial damage in adult cardiomyocytes subjected to sI/R with or without TAT-Ndi1, cytochrome *c* release was monitored by immunofluorescence. 73% of TAT-Ndi1 treated cells retained cytochrome *c* in mitochondria compared to 17% of cardiomyocytes subjected to sI/R in the absence of TAT-Ndi1 (Unt) (Figure 3D). Electron microscopy of adult rat cardiomyocytes subjected to sI/R show a frequent loss of defined mitochondrial crista structure, with only 48% of mitochondria retaining detectable cristae. In cells transduced with TAT-Ndi1, 89% of mitochondria have well-defined invaginations of the cristae, comparable to control cells (no I/R) (Figure 3E, * indicates absence of cristae).

### TAT-Ndi1 localizes to mitochondria in Langendorff-perfused rat hearts

**Figure 4:**
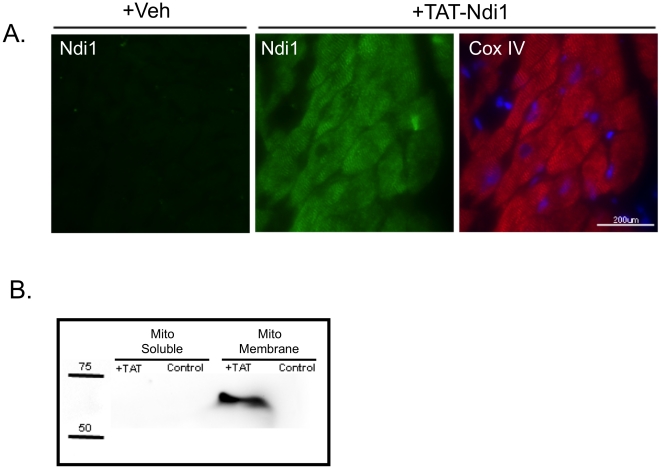
TAT-Ndi1 localization in Langendorff-perfused rat heart. **A**. Cryosections of rat hearts perfused 20 min with TAT-Ndi1 or with vehicle alone (+Veh). Heart sections were stained with rabbit antibody to Ndi1 (green), mouse monoclonal antibody specific for complex IV (red) and Hoechst 33258 nuclear stain (blue). **B**. Hearts were perfused with or without 500nM TAT-Ndi1 for 20 min. Mitochondria were isolated and sonicated to yield membrane and soluble fractions were separated and probed for Ndi1 (representative image).

To confirm localization and the ability to transduce TAT protein by aortic perfusion, Langendorff-perfused rat hearts given vehicle alone or recombinant TAT-Ndi1 were cryosectioned and stained with antibodies to Ndi1 and complex IV. TAT-Ndi1 was found in a striated pattern that co-localized with complex IV (Figure 4A). Mitochondria isolated from Langendorff-perfused rat hearts were subfractionated to generate membrane and soluble protein fractions and probed for Ndi1 expression. TAT-Ndi1 was only detected in the isolated mitochondrial membrane fraction (Figure 4B).

### TAT-Ndi1 overcomes complex I dysfunction due to I/R injury

**Figure 5:**
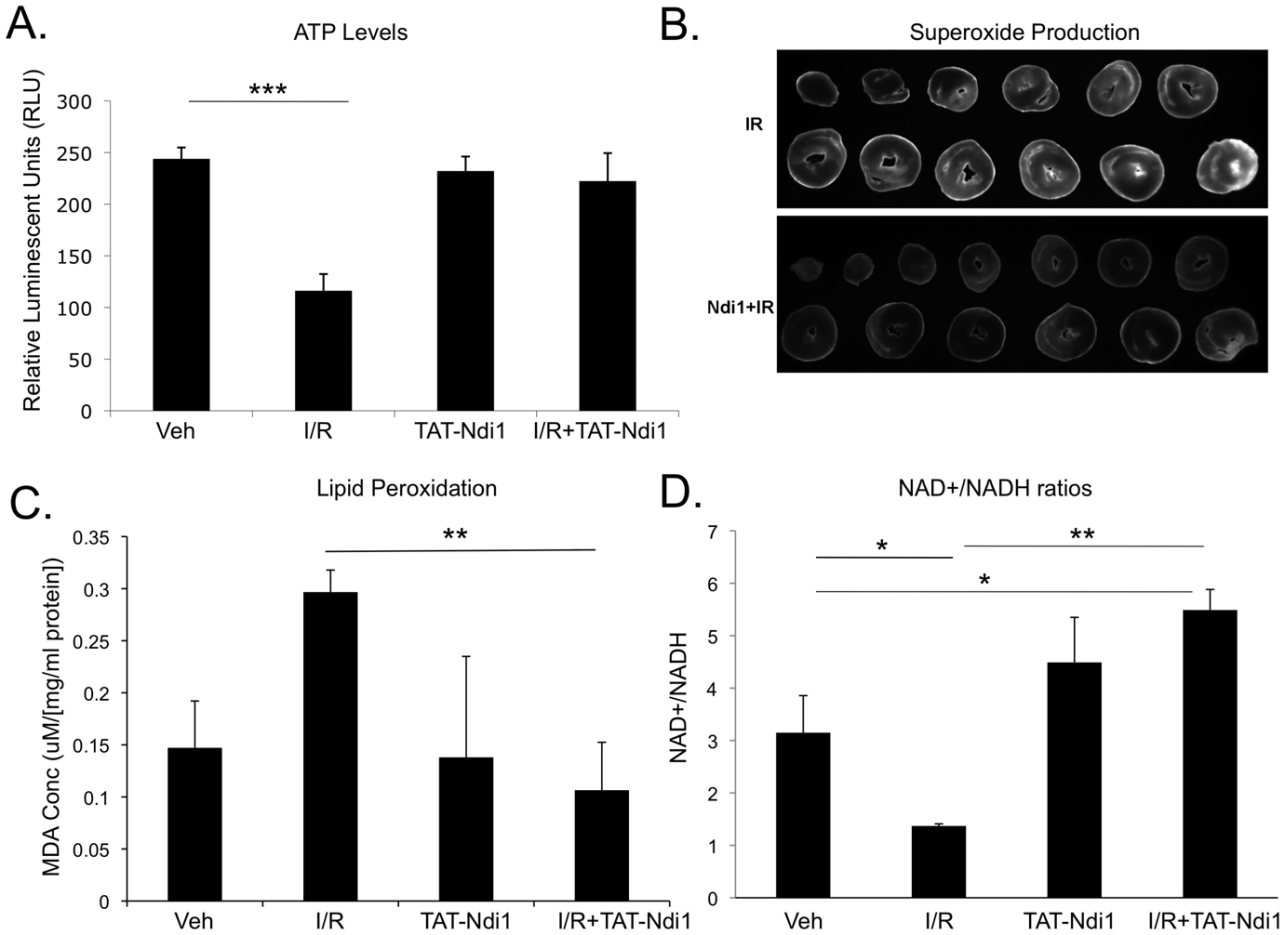
TAT-Ndi1 overcomes effects of complex I dysfunction. **A**. ATP levels in rat heart tissue +/− TAT-Ndi1 following 30 min ischemia and 15min reperfusion or without treatment (veh). TAT-Ndi1 prevents depletion of ATP stores in I/R hearts. (n = 4, *p<0.05, ***p<0.0005). **B**. Dihydroethidium stained 1mm rat heart sections +/−TAT-Ndi1 following 30 min no-flow ischemia and 15 min reperfusion. TAT-Ndi1 reduces superoxide production following I/R (representative image, n = 3). **C**. Total free MDA levels normalized to total protein in hearts perfused 20 min with or without TAT-Ndi1 and subjected to 30 min ischemia and 15 min reperfusion or perfused constantly with vehicle (n = 3, **p<0.005). **D**. NAD^+^/NADH ratios from rat hearts perfused 15 min +/− TAT-Ndi1 then subjected to 30 min ischemia and 15 min reperfusion or perfused continuously with vehicle (n = 4, *p<0.05, **p<0.005).

To determine the effect of TAT-Ndi1 on I/R-induced energetic deficits and oxidative damage, we perfused rat hearts with TAT-Ndi1 followed by global no-flow ischemia and 15 min reperfusion. I/R results in a 50% reduction of ATP content, but TAT-Ndi1 prevents ATP depletion (Figure 5A). Dihydroethidium-stained sections of hearts subjected to I/R showed that TAT-Ndi1 transduction reduced superoxide production (Figure 5B) and lipid peroxidation (Figure 5C). Administration of TAT-Ndi1 shifted the NAD+/NADH ratio towards NAD+ under basal conditions and following I/R. TAT-Ndi1 increased the ratio 3-fold over I/R conditions without TAT-Ndi1 (Figure 5D).

### Mitochondrial integrity and function is preserved by Ndi1 protein transduction

**Figure 6:**
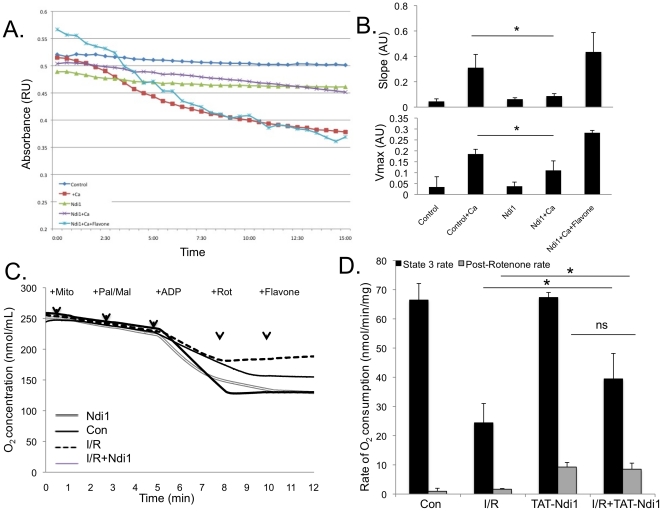
Mitochondrial integrity and function is preserved by TAT-Ndi1. **A**. The absorbance of cardiac mitochondrial suspension from rat heart tissue was measured in the presence or absence of TAT-Ndi1. Hearts were perfused +/− TAT-Ndi1 for 20 min prior to isolating mitochondria. TAT-Ndi1 protects against calcium-induced mitochondrial swelling and this inhibition is abolished by Ndi1 inhibitor, flavone (representative trace, n = 4). **B**. Slope and V_max_ of mitochondrial swelling are reduced in mitochondria with TAT-Ndi1 (n = 4, p<0.05). **C**. Oxygen consumption of mitochondria isolated from rat hearts with or without TAT-Ndi1 and subjected to I/R (I/R+TAT-Ndi1:double line, I/R alone: dashed line) or constantly perfused (Con:thick line, +TAT-Ndi1:thin line). Oxygen levels were continuously monitored using a platinum Clark-type oxygen electrode. Changes of O**_2_** concentration in chamber are shown with administration of treatments indicated (n = 4, representative trace). **D**. Rate of oxygen consumption following addition of complex I substrates palmitoyl-L-carnitine/malate and ADP (1mM final) prior to (black bars) and following (grey bars) addition of rotenone (*p<0.05). Mitochondria were isolated from hearts +/− TAT-Ndi1 subjected to I/R or constantly perfused (control and Ndi1 alone).

Mitochondrial integrity is compromised by I/R injury, often leading to opening of the mitochondrial permeability transition pore (MPTP) and release of pro-apoptotic factors culminating in cell death. ROS generated from complex I are thought to trigger MPTP opening. To determine if TAT-Ndi1 could prevent mitochondrial swelling, hearts were perfused with TAT-Ndi1 or vehicle, then mitochondria were isolated and induced to undergo swelling with the addition of Ca^2+^. TAT-Ndi1 attenuated swelling by 72%±3.0 (slope) and 41%±2.6 (Vmax) (Figure 6A&B). This effect was abolished by inhibition of Ndi1 with flavone. Complex I-dependent respiration was reduced from 66nmol/min/mg to 26nmol/min/mg after I/R, but administration of Tat-Ndi1 increased malate/palmitoyl-L-carnitine-driven oxygen consumption to 40nmol/min/mg after I/R. This improvement can be largely attributed to TAT-Ndi1-dependent respiration, as the rotenone-insensitive rate is 10nmol/min/mg in the presence of TAT-Ndi1, with or without I/R, confirming the functional incorporation of Ndi1 into the respiratory chain. It is possible that Ndi1, through the reduction of ROS levels, also helps to preserve a small amount of complex I activity following I/R.

### TAT-Ndi1 is cardioprotective in the Langendorff-perfused rat heart model of I/R

**Figure 7:**
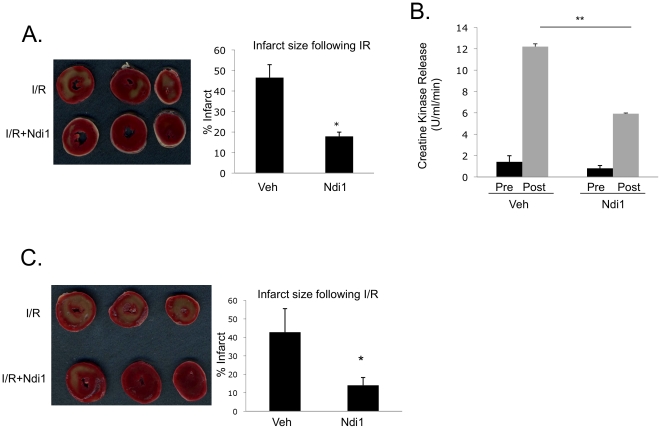
TAT-Ndi1 is cardioprotective in the Langendorff-perfused rat heart model of ischemia/reperfusion. **A**. Rat hearts were perfused with or without TAT-Ndi1 for 20 min prior to 30 min ischemia and 2 hour reperfusion. Frozen sections were stained with TTC. TAT-Ndi1 reduced infarct size 61.5%±8.01. Mean and S.D. from at least 5 hearts per condition. (*p<0.05). **B**. Perfusate collected prior to ischemia (baseline) and 15 min following onset of reperfusion. Creatine kinase release was reduced 51.6%±9.8 following ischemia/reperfusion in hearts perfused with TAT-Ndi1. Mean and S.D. from at least 4 hearts per condition. (**p<0.01). **C**. Hearts were subjected to 30 min ischemia and perfused with or without TAT-Ndi1 at the onset of reperfusion. Hearts were reperfused for 2 hours. Sections were stained with TTC (representative image, n = 5). TAT-Ndi1 reduced infarct size 67.1%±17.1. Mean and S.D. from at least 5 hearts per condition (*p<0.05).

Given the ability of TAT-Ndi1 to overcome the effects of complex I damage and preserve mitochondrial integrity, we wanted to determine the capacity of Ndi1 to protect against I/R injury *ex vivo*. Rat hearts were perfused 20 min with or without TAT-Ndi1 prior to 30 min global no-flow ischemia and 2 hr reperfusion. TAT-Ndi1 reduced infarct size by 62%±8.1, based on TTC staining (Figure 7A). Creatine kinase release was reduced by 51.6%±3.02 following I/R in hearts perfused with TAT-Ndi1 (Figure 7B).

### Ndi1 is protective against I/R injury when administered at the onset of reperfusion

Much of the damage to mitochondria occurs during the early minutes of reperfusion. To determine whether TAT-Ndi1 can protect the heart if given at reperfusion, Ndi1 was added to perfusion buffer at the onset of reperfusion after 30 min ischemia and hearts were reperfused for 2 hours. TTC staining indicated that infarct size was reduced by 58.2%±4.2 when TAT-Ndi1 was administered at reperfusion. This degree of protection is comparable to that observed with pretreatment (Figure 7C).

## Discussion

Ischemia reperfusion injury in the heart is a leading cause of morbidity in the western world. Complexes I, III, IV and V of the respiratory chain and many Krebs cycle enzymes are compromised by I/R injury [28]–[30]. Oxidative damage plays a central role in cardiac dysfunction resulting from I/R, with maximal generation of ROS and reactive nitrogen species occurring at the onset of reperfusion. Complex I is particularly susceptible to oxidative damage and subsequently produces more ROS [31], leading to extensive mitochondrial dysfunction and the depletion of ATP. Complex I dysfunction also impairs the oxidation of NADH, which can lead to the production of superoxide radicals through the FMN group of complex I and α-ketoglutarate dehydrogenase [32]. ROS-induced ROS release leads to MPTP with mitochondrial swelling, release of pro-apoptotic factors such as cytochrome *c* and cell death via apoptosis and necrosis [33]. Thus, complex I plays a central role mediating I/R injury.

Bypassing damaged complex I with a single-subunit enzyme that will oxidize NADH in the matrix addresses the accumulation of NADH and the resulting oxidative damage. The expression of Ndi1, the yeast alternate NADH-quinone oxidoreductase enzyme, prevented cell death in both *in vitro* and *ex vivo* models of I/R, demonstrating that I/R injury in the heart is tied to dysfunction of complex I and oxidative damage. In order to deliver Ndi1 protein to tissue, we generated a TAT-Ndi1 fusion protein capable of entering cells and localizing to the mitochondrial matrix, where it was able to transfer electrons from NADH to ubiquinone. We show here that TAT-Ndi1 favors NADH oxidation following I/R, confirming functional integration of the TAT protein into the host respiratory chain. Maintenance of this redox potential confers protection from oxidative stress and prevents transfer of electrons from NADH to oxygen via damaged complex I.

In comparison to complex I, Ndi1 catalyzes a two-electron transfer reaction that is believed to prevent the formation of an ubisemiquinone intermediate during the process of NADH oxidation [9], [12]. This reaction mechanism minimizes electron leakage and the subsequent formation of ROS. It has been demonstrated that Ndi1-mediated NADH oxidation does not generate superoxide radicals, in distinction to other NDH-2 enzymes and complex I/NDH-1 [34]. In our studies, Ndi1 lowered ROS generation and lipid peroxidation in cells subjected to sI/R and heart tissue following I/R. Coupled with preventing ROS generation by complex I, likely through maintaining a high redox potential (low matrix NADH/NAD+ ratio) [35], [36], Ndi1 is an effective combatant of oxidative damage, especially under I/R conditions.

The cardioprotective effects of Ndi1 were robust, accompanied by a significant reduction in oxidative damage. Preservation of ATP levels was indirect, since Ndi1 does not pump protons and is likely a reflection of less cell death. The ability of Ndi1 to prevent complex I-mediated ROS release rather than effects on ATP production was the most likely mechanism of protection in combination with the effects on the NAD^+^/NADH ratio [36]. Other NAD^+^/NADH-dependent pathways may be directly or indirectly affected by Ndi1-mediated oxidation of NADH under ischemic conditions. For instance, aldehyde dehydrogenases require NAD^+^ as a cofactor in the metabolism of acetylaldehyde and other toxic aldehydes. Mitochondrial aldehyde dehydrogenase (ALDH2) has been implicated in cardioprotection from ischemic injury by both modulating the autophagic pathway and through inhibiting formation of 4-hydroxy-2-nonenal (4-HNE)-protein adducts [40]. It is possible that TAT-Ndi1 helps to maintain ALDH2 activity, thereby reducing toxic aldehyde formation. Caspase-independent apoptotic cell death may also be attenuated by eliminating PARP activation by ROS [41]. Sirtuins are NAD^+^-dependent proteins implicated in defense against aging, diabetes, stress and Alzheimer's Disease. Although sirtuins are regulated by the NAD^+^/NADH ratio, overexpression of Ndi1 in Drosophila did not increase Sir2 activity although fly lifespan was extended by 20–40%, an effect attributed to diminished mitochondrial ROS production [42].

TAT-mediated protein transduction provides a novel and efficient approach to address complex I deficiencies and is a burgeoning area of interest in the delivery of a wide range of therapeutic molecules. TAT-fusion proteins and peptides have been used to promote cell death in mouse cancer models including prostate, breast, leukemia, melanoma, and glioma [37]. A TAT-fusion peptide inhibitor of protein kinase C delta (KAI-9803) showed promise in a clinical trial of myocardial infarct size reduction [38]. Additional clinical trials are underway for treatment of ischemic stroke and neurodegenerative disease using KAI-9803 [39]. Effective replacement therapy for complex I deficiencies and protection from I/R injury requires efficient incorporation of the enzyme into the host respiratory chain. Treatment of I/R injury, in particular, requires rapid incorporation during the first minutes of reperfusion. We have shown that TAT-Ndi1 functionally incorporated into host mitochondria and conferred cardioprotection when administered either as a pretreatment or at the onset of reperfusion, indicating rapid protein transduction. This is the first time functional delivery of a mitochondrial inner membrane-targeted TAT protein has been demonstrated and our results show that Ndi1 has significant protective effects on mitochondrial integrity and overall oxidative state.

## References

[1] Hirst J, Carroll J, Fearnley IM, Shannon RJ, Walker JE (2001). The nuclear encoded subunits of complex I from bovine heart
mitochondria.. Biochim Biophys Acta.

[2] Walker JE (1992). The NADH:ubiquinone oxidoreductase (complex I) of respiratory
chains.. Q Review Biophys.

[3] Janssen RJ, Van den Heuvel LP, Smeitink JA (2004). Genetic detects in the oxidative phosphorylation (OXPHOS)
system.. Expert Rev Mol Diagn.

[4] Ambrosio G, Zweier JL, Flaherty JT (1991). The relationship between oxygen radical generation and impairment
of myocardial energy metabolism following post-ischemic
reperfusion.. J Mol Cell Cardiol.

[5] Hess ML, Manson NH (1994). Molecular oxygen: friend and foe. The role of the oxygen free
radical system in the calcium paradox, the oxygen paradox and
ischemia/reperfusion injury.. J Mol Cell Cardiol.

[6] Das DK (1994). Cellular biochemical and molecular aspects of reperfusion
injury.. Ann N Y Acad Sci.

[7] Zorov DB, Filburn CR, Klotz LO, Zweier JL, Sollott SJ (2000). Reactive oxygen species (ROS)-induced ROS release: A new
phenomenon accompanying induction of the mitochondrial permeability
transition in cardiac myocytes.. J Exp Med.

[8] Yagi T (1991). Bacterial NADH-quinone oxidoreductase.. J Bioeng Biomem.

[9] de Vries S, Grivell LA (1988). Purification and characterization of a rotenone-insensitive
NADH:Q6 oxidoreductase from mitochondria of Saccharomyces
cerevisiae.. Eur J Biochem.

[10] Marres CAM, de Vries S, Grivell LA (1991). Isolation and inactivation of the nuclear gene encoding the
rotenone-insensitive internal NADH: ubiquinone oxidoreductase of
mitochondria from Saccharomyces cerevisiae.. Eur J of Biochem.

[11] de Vries S, Van Witzenburg R, Grivell LA, Marres CAM (1992). Primary structure and import pathway of the rotenone-insensitive
NADH-ubiquinone oxidoreductase of mitochondria from Saccharomyces
cerevisiae.. Eur J of Biochem.

[12] Kitajima-Ihara T, Yagi T (1998). Rotenone-insensitive internal NADH-quinone oxidoreductase of
Saccharomyces cerevisiae mitochondria: the enzyme expressed in Escherichia
coli acts as a member of the respiratory chain in the host
cells.. FEBS Letters.

[13] Seo BB, Wang J, Flotte TR, Yagi T, Matsuno-Yagi A (2000). Use of the NADH-quinone oxidoreductase (NDI1) gene of
Saccharomyces cerevisiae as a possible cure for complex I defects in human
cells.. J Biol Chem.

[14] Seo BB, Kitajima-Ihara T, Chan EK, Scheffler IE, Matsuno-Yagi A (1998). Molecular remedy of complex I defects: Rotenone-insensitive
internal NADH-quinone oxidoreductase of Saccharomyces cerevisiae
mitochondria restores the NADH oxidase activity of complex I-deficient
mammalian cells.. Proc Natl Acad Sci USA.

[15] Seo BB, Nakamaru-Ogiso E, Flotte TR, Matsuno-Yagi A, Yagi T (2006). In vivo complementation of complex I by the yeast Ndi1 enzyme:
possible application for treatment of Parkinson disease.. J Biol Chem.

[16] Claycomb WC, Lanson NA, Stallworth BS, Egeland DB, Delcarpio JB (1998). HL-1 cells: a cardiac muscle cell line that contracts and retains
phenotypic characteristics of the adult cardiomyocyte.. Proc Natl Acad Sci U S A.

[17] Iwaki K, Sukhatme VP, Shubeita HE, Chien KR (1990). Alpha- and beta-adrenergic stimulation induces distinct patterns
of immediate early gene expression in neonatal rat myocardial cells. fos/jun
expression is associated with sarcomere assembly; Egr-1 induction is
primarily an alpha 1-mediated response.. J Bio Chem.

[18] He H, Li HL, Lin A, Gottlieb RA (1999). Activation of the JNK pathway is important for cardiomyocyte
death in response to simulated ischemia.. Cell Death Differ.

[19] Chen M, Won DJ, Krajewski S, Gottlieb RA (2002). Calpain and mitochondria in ischemia/reperfusion
injury.. J Biol Chem.

[20] Granville DJ, Tashakkor B, Takeuchi C, Gustafsson AB, Huang C (2004). Reduction of ischemia and reperfusion-induced myocardial damage
by cytochrome P450 inhibitors.. Proc Natl Acad Sci USA.

[21] Miller FJJ, Gutterman DD, Rios CD, Heistad DD, Davidson BL (1998). Superoxide production in vascular smooth muscle contributes to
oxidative stress and impaired relaxation in atherosclerosis.. Circ Res.

[22] Sayen MR, Gustafsson AB, Sussman MA, Molkentin JD, Gottlieb RA (2003). Calcineurin transgenic mice have mitochondrial dysfunction and
elevated superoxide production.. Am J Physiol Cell Physiol.

[23] Pain T, Yang XM, Critz SD, Yue Y, Nakano A (2000). Opening of mitochondrial KATP channels triggers the
preconditioned state by generating free radicals.. Circ Res.

[24] Becker-Hapak M, Dowdy SF (2003). Protein transduction: generation of full-length transducible
proteins using the TAT system.. Curr Protoc Cell Biol Chapter.

[25] Gustafsson AB, Sayen MR, Williams SD, Crow MT, Gottlieb RA (2002). TAT protein transduction into isolated perfused hearts:
TAT-apoptosis repressor with caspase recruitment domain is
cardioprotective.. Circulation.

[26] Gustafsson AB, Gottlieb RA, Granville DJ (2005). TAT-mediated protein transduction: delivering biologically active
proteins to the heart.. Methods Mol Med.

[27] Becker-Hapak M, McAllister SS, Dowdy SF (2001). TAT-mediated protein transduction into mammalian
cells.. Methods.

[28] Paradies G, Petrosillo G, Pistolese M, Di Venosa N, Serena D (1999). Lipid peroxidation and alterations to oxidative metabolism in
mitochondria isolated from rat heart subjected to ischemia and
reperfusion.. Free Rad Biol Med.

[29] Lesnefsky EJ, Slabe TJ, Stoll MS, Minler PE, Hoppel CL (2001). Myocardial ischemia selectively depletes cardiolipin in rabbit
heart subsarcolemmal mitochondria.. Am J Physiol Heart Circ Phys.

[30] Petrosillo G, Ruggiero FM, Di Venosa N, Paradies G (2003). Decreased complex III activity in mitochondria isolated from rat
heart subjected to ischemia and reperfusion: role of reactive oxygen species
and cardiolipin.. FASEB J.

[31] Lesnefsky EJ, Chen Q, Moghaddas S, Hassan MO, Tandler B (2004). Blockade of electron transport during ischemia protects cardiac
mitochondria.. J Biol Chem.

[32] Kussmaul L, Hirst J (2006). The mechanism of superoxide production by NADH:ubiquinone
oxidoreductase (complex I) from bovine heart mitochondria.. Proc Natl Acad Sci U S A.

[33] Lesnefsky EJ, Tandler B, Ye J, Slabe TJ, Turkaly J (1997). Myocardial ischemia decreases oxidative phosphorylation through
cytochrome oxidase in subsarcolemmal mitochondria.. Am J Physiol.

[34] Fang J, Beattie DS (2003). External alternative NADH dehydrogenase of *Saccharomyces
cerevisiae*: a potential source of superoxide.. Free Rad Bio Med.

[35] Marella M, Seo BB, Matsuno-Yagi A, Yagi T (2007). Mechanism of cell death caused by complex I defects in a rat
dopaminergic cell line.. J Biol Chem.

[36] Seo BB, Marella M, Yagi T, Matsuno-Yagi A (2006). The single subunit NADH dehydrogenase reduces generation of
reactive oxygen species from complex I.. FEBS Lett.

[37] Kim CH, Woo SJ, Park JS, Kim HS, Park MY (2007). Enhanced antitumour immunity by combined use of temozolomide and
TAT-survivin pulsed dendritic cells in a murine glioma.. Immunology.

[38] Bates E, Bode C, Costa M, Gibson CM, Granger C (2008). Intracoronary KAI-9803 as an adjunct to primary percutaneous
coronary intervention for acute ST-segment elevation myocardial
infarction.. Circulation.

[39] Bright R, Steinberg GK, Mochly-Rosen D (2007). DeltaPKC mediates microcerebrovascular dysfunction in acute
ischemia and in chronic hypertensive stress in vivo.. Brain Res.

[40] Ma H, Guo R, Yu L, Zhang Y, Ren J (2010). Aldehyde dehydrogenase 2 (ALDH2) rescues myocardial
ischaemia/reperfusion injury: role of autophagy paradox and toxic
aldehyde.. Eur Heart J.

[41] Siegel C, McCullough LD (2010). NAD+ Depletion or PAR Polymer Formation: Which Plays the
Role of Executioner in Ischemic Cell Death?. Acta Physiol.

[42] Sanz A, Soikkeli M, Portero-Otin M, Wilson A, Kemppainen E (2010). Expression of the yeast NADH dehydrogenase Ndi1 in
*Drosophila* confers increased lifespan independently of
dietary restriction.. PNAS.

